# Poly(lactic-co-glycolic) Acid/Nanohydroxyapatite Scaffold Containing Chitosan Microspheres with Adrenomedullin Delivery for Modulation Activity of Osteoblasts and Vascular Endothelial Cells

**DOI:** 10.1155/2013/530712

**Published:** 2013-06-11

**Authors:** Lin Wang, Chunyan Li, Yingxin Chen, Shujun Dong, Xuesi Chen, Yanmin Zhou

**Affiliations:** ^1^VIP Integrated Department, School of Stomatology, Jilin University, 1500 Qinghua Road, Changchun 130021, China; ^2^Implant Center, School of Stomatology, Jilin University, Changchun 130021, China; ^3^State Key Laboratory of Polymer Physics and Chemistry, Changchun Institute of Applied Chemistry, Chinese Academy of Sciences, 5625 Renmin Avenue, Changchun 130022, China

## Abstract

Adrenomedullin (ADM) is a bioactive regulatory peptide that affects migration and proliferation of diverse cell types, including endothelial cells, smooth muscle cells, and osteoblast-like cells. This study investigated the effects of sustained release of ADM on the modulation activity of osteoblasts and vascular endothelial cells *in vitro*. Chitosan microspheres (CMs) were developed for ADM delivery. Poly(lactic-co-glycolic) acid and nano-hydroxyapatite were used to prepare scaffolds containing microspheres with ADM. The CMs showed rough surface morphology and high porosity, and they were well-distributed. The scaffolds exhibited relatively uniform pore sizes with interconnected pores. The addition of CMs improved the mechanical properties of the scaffolds without affecting their high porosity. *In vitro* degradation tests indicated that the addition of CMs increased the water absorption of the scaffolds and inhibited pH decline of phosphate-buffered saline medium. The expression levels of osteogenic-related and angiogenic-related genes were determined in MG63 cells and in human umbilical vein endothelial cells cultured on the scaffolds, respectively. The expression levels of osteogenic-related and angiogenic-related proteins were also detected by western blot analysis. Their expression levels in cells were improved on the ADM delivery scaffolds at a certain time point. The *in vitro* evaluation suggests that the microsphere-scaffold system is suitable as a model for bone tissue engineering.

## 1. Introduction

Bone regeneration comprises a well-orchestrated series of biological events including the recruitment and proliferation of osteoprogenitors from mesenchymal stem cells, cell differentiation, osteoid formation, and ultimately mineralization [1]. Complex clinical conditions in which bone regeneration materials are required in large quantity exist. Scaffolds for bone engineering are degradable matrices designed to support cell adhesion, proliferation, and differentiation for bone regeneration.

The Food and Drug Administration for human biomedical applications approved the use of poly(lactic-co-glycolic) acid (PLGA) as a commercially available biomaterial [2]. PLGA is relatively hydrophobic and obstructs cell adhesion, which are the common weaknesses for synthetic polymers. Nevertheless, it can form scaffolds with high mechanical strength. Similar to natural bone mineral, hydroxyapatite (HA) is relatively easier to be identified by cells or biomacromolecules, which can improve the bioactivity, bioavailability, and biocompatibility of scaffolds. Moreover, the release of calcium and phosphorus ions during the degradation of HA may be involved in bone metabolism to promote the formation of new bone. The combination of PLGA with natural polymers, such as HA, might overcome the limitations of synthetic and naturally derived polymers alone and produce a material with properties (e.g., high porosity and controllability of pore size) beneficial for biomedical applications [3] to mimic bone structure and substructure.

The continued enhancement of biomaterial strategies is known to be highly dependent on the ability to promote rapid and stable vascularization within scaffolds [4]. The most common approach for vascularization in biomaterials emerged to address biological growth factors into scaffolds, thus paving the way for vascular endothelial cell seeding and polymer bioactivity [5]. Adrenomedullin (ADM), a 52-amino acid ringed-structure peptide with C-terminal amidation, is a newly discovered member of the calcitonin peptide family; it was originally isolated from human pheochromocytoma [6]. ADM is also present in many other tissues, such as bone [7], kidney, lung, heart, and adrenals [8]. Many studies focused on the cardiovascular and endocrine effects of ADM [9–11]. However, in recent years, other significant effects, such as osteogenesis [12], angiogenesis [13], and antibacterial effects [14, 15], have also been detected. Many studies [16, 17] showed that ADM can stimulate osteoblast proliferation even at low concentrations. Given its structural and biological homology with calcitonin gene-related peptide, ADM can also stimulate the proliferation of osteoblasts by increasing the cAMP level in osteoblast-like cells [18, 19]. In addition, ADM has a regulatory function in angiogenesis by modulating endothelial cell behavior. However, similar to the dilemma of peptides in treating bone defects, the application of ADM not only requires appropriate temporary release but also requires a certain concentration to be sustained through controlled release during bone regeneration. An efficient delivery system may be required to provide the controlled release of ADM over an extended period.

To date, a couple of biodegradable polymers have been used to encapsulate proteins and peptides. Chitosan or poly *β*-(1,4)-2-amino-2-deoxy-D-glucose is an excellent natural hydrophilic cationic polysaccharide derived from chitin. It is widely used for the controlled delivery of polypeptides and proteins in the form of microspheres or nanospheres [20]. Chitosan exhibits favorable biological properties, such as biodegradability, biocompatibility, nontoxicity, hemostaticity, high surface-charge density, bacteriostaticity, and strong adhesion [21]. It is also used in the field of surgical sutures, wound dressings, drug delivery agents, defect fillers, and tissue-engineering scaffolds [22]. Simple adsorption of growth factors into chitosan allows local delivery, but the temporal control over release kinetics is limited [23]. An ideal delivery system can be designed as chitosan microspheres (CM) compound with porous scaffolds of proper mechanical properties. These two components can coordinately enhance tissue regeneration and extend the release time of growth factors.

Previous studies improved the bioactivity of scaffolds by coating some proteins or peptides to receive and respond to specific biological signals. In this study, CMs loaded with ADM were prepared by an emulsion-ionic cross-linking method. CMs were embedded in a PLGA/nanohydroxyapatite (nHA) scaffold to enhance the compressive strength and develop a microsphere-scaffold system with the capacity of releasing bioactive factor in a well-controlled manner. The *in vitro* ADM release kinetics of microspheres and composite scaffolds was demonstrated in our previous study [24]. The present study aims to investigate the feasibility of using CMs as a carrier for the controlled release of regulatory peptide ADM. The surface morphology, size distribution, and encapsulation efficiency (EE) of the microspheres were estimated. After introducing CMs with ADM into PLGA/nHA scaffolds, the morphological and mechanical features and degradation behavior of the composite scaffolds were evaluated. The biological capabilities of the PLGA/nHA scaffolds were evaluated by culturing MG63 cells and human umbilical vein endothelial cells (HUVEC) on these scaffolds by real-time polymerase chain reaction (PCR) and western blot analysis.

## 2. Materials and Methods

### 2.1. Materials

Chitosan (*M*_*w*_ = 500 kDa) was purchased from Jinqiao Chemical Reagents Company (Taizhou, Zhejiang, China). Human ADM (purity = 95% by HPLC) was obtained from Phoenix Pharmaceuticals (Burlingame, Canada). Tripolyphosphate (TPP) and span-80 were obtained from Aladdin (Shanghai, China). PLGA (nLA/nGA = 80/20) and nHA were obtained from Changchun Institute of Applied Chemistry Chinese Academy of Sciences. MG63 and HUVEC cells were provided by the Basic Medical Collage of Jilin University. Liquid paraffin, 1,4-dioxane, phosphate-buffered saline (PBS, pH 7.4), and other chemicals were all analytical grade and used as received.

### 2.2. Preparation and Characterization of TPP-CMs

Using TPP as cross-linker, CMs loaded with ADM were prepared by an emulsion-ionic cross-linking method. Briefly, 900 mg of chitosan was dissolved in 29 mL of 2% (v/v) aqueous acetic acid and stirred until the solution was transparent. Meanwhile, 500 *μ*g of ADM was dissolved in 1 mL of 2% (v/v) acetic acid and added into the chitosan solution. The mixture was poured into 300 mL of liquid paraffin containing 2% (w/v) of span-80 and stirred mechanically for 2 h. Then, 70 mL of 5% (w/v) TPP was dropped into the emulsion and stirred for 4 h at room temperature. The end emulsion was repeatedly washed with excess amounts of petroleum ether, isopropyl alcohol, and distilled water. The microspheres were obtained after lyophilization (LGJ-18, Sihuan, China).

The morphology of the CMs was examined under a scanning electron microscope (SEM; XL30ESEM-FEG, FEL, Netherlands). For the measurement, the microspheres were attached to metal stubs and sputter coated with gold under vacuum. In addition, the diameter of the microspheres was determined by a laser particle size analyzer (LS 13 320, Beckman Coulter, USA).

A certain amount of ADM-loaded chitosan microspheres (CMs-ADM) was dissolved in 5 mL of 2% aqueous acetic acid solution and filtered to remove any undissolved residue. The amounts of ADM in the collected supernatants were measured by HPLC. Encapsulated efficiency (EE) was calculated as follows using the data above:

(1) $$\begin{array}{c} \text{EE}\left(\%\right)=\frac{\text{actual}\;\text{ADM}\;\text{amount}}{\text{theoretical}\;\text{ADM}\;\text{amount}}\times 100\%. \end{array}$$

All measurements were performed in triplicate for each of the samples.

### 2.3. Preparation and Characterization of Scaffolds

Porous PLGA/nHA/CMs scaffolds were developed by thermally induced phase separation (TIPS). PLGA (720 mg) was dissolved in 12 mL of 1,4-dioxane, and nHA (360 mg) was added into the mixture after stirring for half an hour. Ultrasonics was used to completely disperse the nHA for 10 min. Then, 240 mg of CMs was added to the aforementioned solution. The mixture was agitated by magnetic stirring to completely disperse the microspheres and poured into a polytetrafluoroethene plate. Then, the solution was frozen overnight in a refrigerator at −20°C. Finally, PLGA/nHA/CM scaffolds were obtained after lyophilization. Pure PLGA/nHA scaffolds prepared by the same method were set as a control for the succeeding experiments.

The pore architecture of the scaffolds, which were located on the metal stubs and sputter coated with gold, was examined by SEM. The porosity of the scaffolds was measured by a mercury intrusion porosimeter (AutoPore IV 9500, USA). Percent porosity was provided in the output from the equipment. In addition, the density of the polymer scaffolds was tested by a modified liquid displacement method [25].

### 2.4. Test of Mechanical Properties

The resistance to mechanical compression of the scaffolds was tested on an electromagnetic testing machine (Enduratec Elf 3200, Bose Corporation, Eden Prairie, MN, USA) with a 10 kN load cell at room temperature. The samples were cylinders with a diameter of 8 mm and a height of 15 mm. Compression tests were carried out under displacement control at a velocity of 0.1 mm/s until the sample was 50% of the initial height. The compressive modulus and compressive strength were calculated as the average of three scaffold measurements.

### 2.5. *In Vitro* Degradation Test

 The cylinder scaffolds with a diameter of 8 mm and a height of 5 mm were incubated at 37°C in 10 mL PBS, pH 7.4. The samples were centrifuged for 3 min to ensure that the entire scaffold was immersed into the buffer, except in water absorption (WA) test, and then incubated at 37°C under dynamic conditions for 12 weeks. The incubation buffer was weekly replaced with fresh PBS solution, except in pH changing test. At scheduled fold (once a week), the samples were washed with distilled water and lyophilized. The pH of the PBS solution during degradation was monitored by a pH meter (FE20, Mettler Toledo, Shanghai, China).

 All data presented in the figures of this paper are the average data from six parallel samples.

#### 2.5.1. Weight Loss (WL)

The WL of scaffolds was gravimetrically examined (AL 104, Mettler Toledo, Shanghai, China) at scheduled times after the samples were freeze dried. WL was computed as follows:

(2) $$\begin{array}{c} \text{WL}\left(\%\right)=\frac{W_{0}-W_{t}}{W_{0}}\times 100\%,\;\left(n=6\right), \end{array}$$

where *W*_0_ and *W*_*t*_ are the weights of the samples before and after incubation, respectively.

#### 2.5.2. WA Property

 The initial weight of the dry sample was characterized as *W*_0_′. The scaffolds were taken out from PBS at intervals and gravimetrically weighed (Mettler Toledo AL 104) after wiping off the surface water to obtain the wet mass *W*_*t*_′. Water content was computed as follows:

(3) $$\begin{array}{c} \text{WA}\left(\%\right)=\frac{W_{t}^{\prime }-W_{0}^{\prime }}{W_{t}^{\prime }}\times 100\%,\;\left(n=6\right). \end{array}$$

#### 2.5.3. pH of Degradation Medium

The pH of the degradation medium was measured using a pH meter once a week for 12 weeks. The medium was not refreshed in the entire degradation period.

### 2.6. Gene and Protein Expression of MG63 and HUVEC Cells Cultured on the Scaffolds

We determined the expression levels of osteogenic-related (osteopontin (opn), runt-related transcription factor 2 (runx2), transcription factor 7 (sp7), and collagen type 1 (col1)) and angiogenic-related (vascular endothelial growth factor (vegf) and G-protein coupled activity-modifying protein 2 (ramp2)) genes in MG63 and HUVEC cells, respectively. Meanwhile, the expression levels of osteogenic-related (RUNX2 and COLLAGEN-1) and angiogenic-related (VEGF) proteins were determined in MG63 and HUVEC cells, respectively. The polymer scaffolds with and without CMs-ADM were prepared as cylinders with a diameter of 10 mm and a height of 2 mm and then sterilized with a 25 kGy Co^60^ radiation in preparation for cell seeding.

MG63 and HUVEC cell lines were, respectively, maintained in Dulbecco's modified Eagle's medium (Sigma, ST. Louis, MO, USA) and Iscove's modified Dulbecco's medium (Sigma, ST. Louis, MO, USA) containing 10% fetal bovine serum in an incubator with humidified atmosphere containing 95% air and 5% CO_2_. For subculture, cells at 80% to 90% confluence were passaged at a ratio of 1 : 3 after treating with 0.25% trypsin. The sterilized scaffolds were seeded with approximately 1.5 × 10^5^ cells of the third passage. The cell-scaffold complexes were cultured in 12-well tissue culture plates for up to 5 d. The cells were retrieved for gene and protein expression determination at specific time points (days 1, 3, and 5). The cells cultured in normal condition without scaffold were selected as control.

#### 2.6.1. Quantitative Real-Time PCR

Total RNA was isolated from retrieved MG63 and HUVEC cells of different time points using the Trizol reagent (Invitrogen, Carlsbad, Canada) according to the manufacturer's protocol. The purity of RNA was determined at 260 and 280 nm absorbance to ensure that the ratio was beyond 1.8. Reverse transcription was performed with a Quantscript RT Kit (Tiangen, Beijing, China) using 1 *μ*g total RNA to obtain cDNA. Real-time quantitative PCR was performed for the quantification of gene expression using a Realtime SYBR Green I PCR Master Mix (TOYOBO, Japan) in a StepOnePlus Real-Time PCR System (ABI, Foster City, CA, USA).  Table 1 lists the primers designed by Premier Primer 5 and checked by BLAST. The relative expression levels of genes were analyzed using the 2^−ΔΔCt^ method [26] by normalizing with GAPDH expression and presented as fold increase relative to the control group.

#### 2.6.2. Western Blot Analysis

MG63 and HUVEC cells were retrieved at predetermined time points, washed with ice-cold PBS, and centrifuged at 5000 ×g for 5 min at 4°C. Then, the cells were lysed in ice-cold lysis buffer (10 mM Tris, pH 7.4, 100 mM NaCl, 1 mM ethylenediaminetetraacetic acid, 1 mM phenylmethanesulfonyl fluoride, 1% Triton X-100, 10% glycerol, 0.1% sodium dodecyl sulfate (SDS), and 0.5% deoxycholate) on ice for 1 h. After centrifugation, protein concentration was determined using a NanoDrop ND1000 (ThermoScientific, Wilmington, DE, USA) spectrophotometer. The samples (40 *μ*g protein) were resolved by 10% SDS-polyacrylamide gel electrophoresis and electrophoretically transferred to Immun-Blot polyvinylidene difluoride membranes (Millipore, Bedford, MA, USA). After blocking in Tris-buffered saline with 0.05% Tween-20 (TBST) containing 5% nonfat dry milk for 1 h, the membranes were washed thrice with TBST at room temperature. Then, primary antibodies (SantaCruz Biotechnology, Santa Cruz, CA, USA) were added on the membranes and incubated overnight at 4°C. After incubation with the appropriate horseradish peroxidase, protein bands coupled with secondary antibody (1 : 5000 dilution; Proteintech Group, Chicago, USA) were visualized with an enhanced chemiluminescent system. Protein levels from immunoblot were quantified by densitometry using Quantity One software (Bio-Rad, USA). Target proteins were normalized against *β*-actin expression.

### 2.7. Statistical Analysis

All data were expressed as mean ± SD. Statistical significance of differences was assessed by one-way ANOVA and Student's *t*-test. Statistical significance was considered at *P* < 0.05.

## 3. Results and Discussion

### 3.1. Characterization of TPP-CMs and Polymer Scaffolds

**Figure 1:**
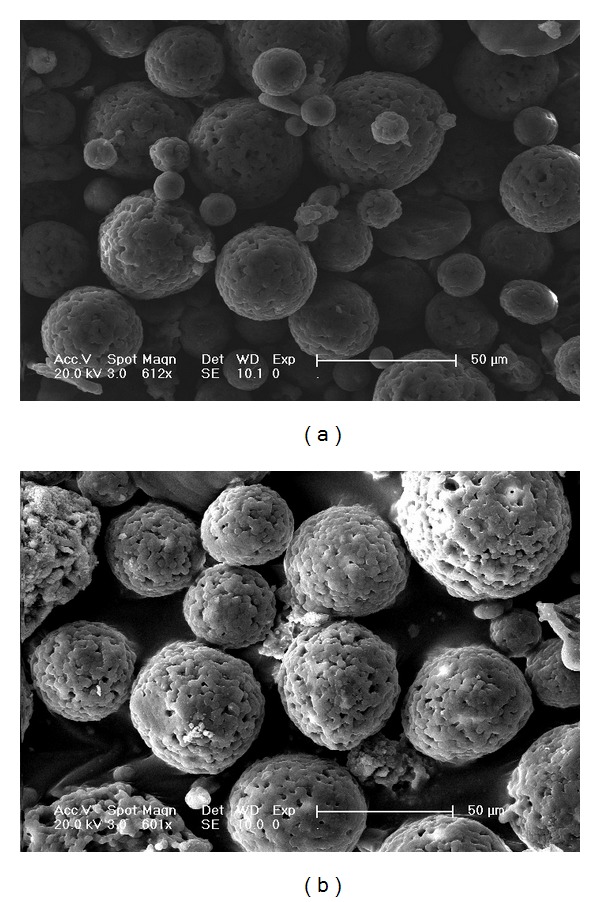
SEM images of chitosan microspheres prepared without (a) and with (b) ADM.

**Figure 2:**
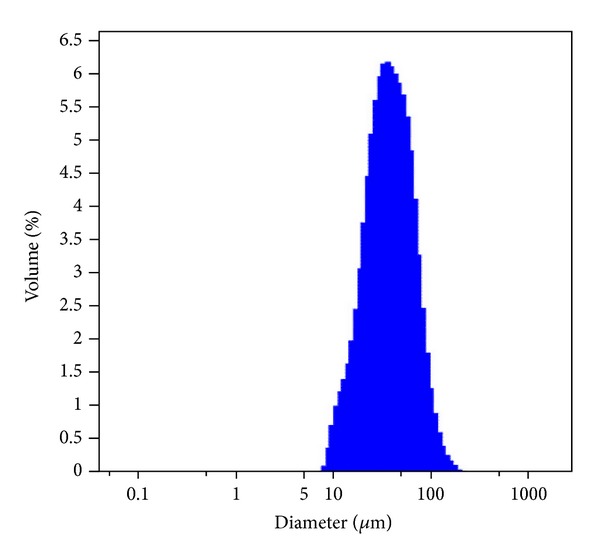
Size distribution of TPP-chitosan microspheres loaded with ADM.

An emulsion-ionic cross-linking method was used in preparing the CMs loaded with ADM in the presence of TPP. The sharp and relative rough surface of TPP-CMs is shown in Figure 1. Microsphere cracks were seldom observed in this study. As shown in Figure 2, the size of the microspheres was well distributed. The average diameter of CMs was 42.69 *μ*m, and EE was 79.4% ± 2.3%. Chitosan was selected for developing microspheres because of its well-known biocompatibility, biodegradability, low toxicity, and low cost [27]. Proteins and peptides released from CMs can be controlled by cross-linking the matrix using chemical cross-linking agents, such as glutaraldehyde, NaOH, and ethylene glycol diglycidyl ether [28]. Ionic cross-linking agents have been developed to avoid the negative effects of chemical cross-linking agents for proteins and peptides [29, 30]. TPP, a nontoxic and multivalent anion, is widely used as an ionic cross-linking agent in the pharmaceutical industry [31]. Polyelectrolyte complex can be formed by ionic interaction between positively charged amino groups of chitosan and multivalent negatively charged TPP molecules under mild conditions [32, 33]. EE is of significant importance for controlled delivery. The stable entrapment of ADM into CMs was achieved by cross-linking the charge and physical interactions. The isoelectric point of ADM is approximately 5.1. Therefore, it carries a negative charge in PBS during the formation of CMs. The ADM can be completely reacted with positively charged chitosan, leading to high EE.

**Figure 3:**
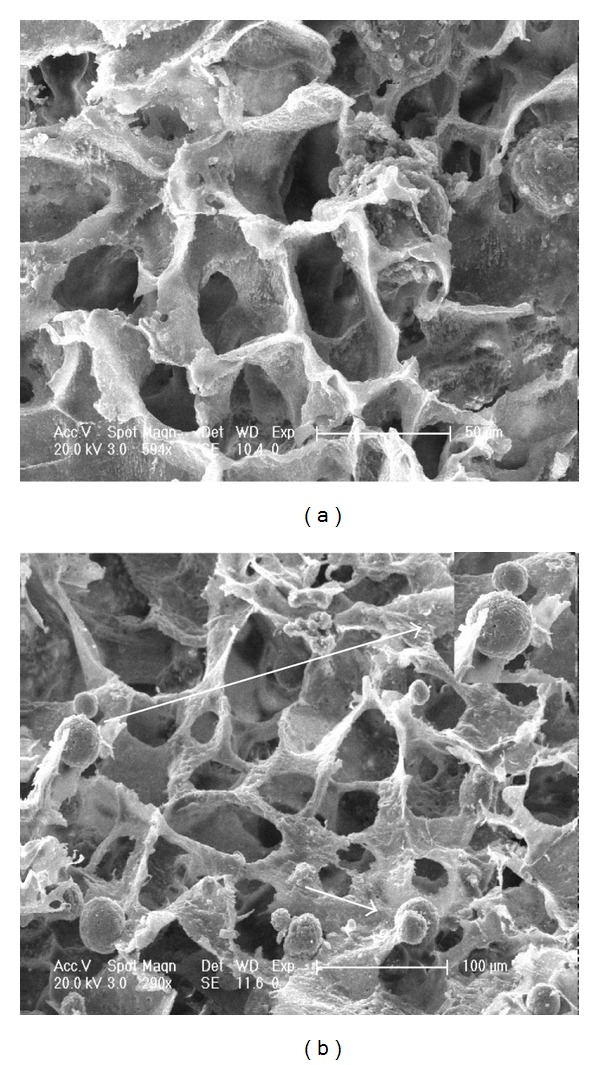
SEM images of PLGA/nHA scaffolds prepared without (a) and with (b) chitosan microspheres. The arrows show the chitosan microspheres in the scaffold.

The porous structure of the scaffolds prepared by TIPS is shown in Figure 3(a). Lactide-based scaffolds made by TIPS can host different types of cells because of their multiscale porosity that supports cell-matrix interactions [34]. The pore size determines cell-seeding efficiency into the scaffold: very small pores prevent the cells from penetrating into the scaffold, whereas very large pores prevent cell attachment because of a reduced area to be colonized by cells [35]. The opened and interconnected pores exhibited a uniform size. In addition, most of these pores were located between 50 and 220 *μ*m, which were suitable for cell and tissue penetration. Scaffolds with micro- and nano-sized architecture similar to that of native bone are important. An ideal scaffold for clinical applications should structurally and functionally mimic native extracellular matrix (ECM) as closely as possible [36]. The substructure of natural bone is composed of nHA and collagen fibers. In this study, nHA mimics the nanostructure of natural bone. 1,4-Dioxane, as a pore-forming agent, was used in the formation of densely packed vertical arrays of dioxane crystals by TIPS, which dominated the final pore structure of the system, resulting in interconnected pore architecture [34]. As shown in Figure 3(b), chitosan microspheres were successfully loaded and well distributed in the polymer scaffolds. The scaffold morphology slightly changed after the introduction of CMs, inferring that the addition of microspheres did not damage the structure of the scaffold. The microspheres in the scaffold can be regarded as some “islands,” which facilitate the adhesion and proliferation of some cells [37, 38].  Table 2 shows the density and porosity of the scaffolds. With the addition of CMs, the density of the scaffolds significantly increased from 0.045 ± 0.017 g/mL to 0.083 ± 0.020 g/mL (*P* < 0.05). However, the decrease in porosity was not significant (from 90.81% ± 0.87% to 88.93% ± 0.32%). The more dense structure of the PLGA/nHA/CM scaffolds was possible because the CMs occupied the available spaces in the prepared scaffolds. In this study, 30% dosage of CMs did not noticeably change the porosity of the scaffold. The addition of 30% CMs in the porous scaffold did not distinctly change the porosity. However, increasing the CM content by up to 50% can apparently decrease the porosity of the composites [39]. Theoretically, the porosity should be influenced by the amount of the added microspheres. Nevertheless, Huang et al. [40] demonstrated that no significant difference in porosity exists even after the addition of 50% microspheres. In bone tissue engineering, scaffolds must have sufficient porosity for nutrient and gas exchange [41]. Satisfactory porosity of more than 80% is a distinct symbol of a perfect scaffold [39]. The porosity of PLGA/nHA polymer scaffolds with and without CMs was all beyond 80%. The high porosity of scaffolds may be a result of an interconnected 3D pore structure. Furthermore, the retention of approximately 90% porosity in the scaffolds should enable a large space for the accommodation of high-density cell cultures.

### 3.2. Mechanical Properties

**Figure 4:**
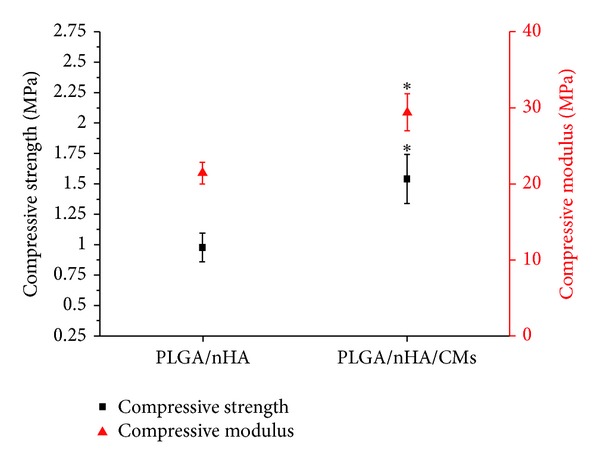
Compressive strength and compressive modulus of PLGA/nHA with and without 30% CMs. *P < 0.05 indicates statistically significant difference compared with the PLGA/nHA group.

Mechanical properties of the PLGA/nHA scaffolds with and without CMs were evaluated by a universal material testing machine. The mechanical parameters of the scaffolds are summarized in Figure 4. The compressive strength of the PLGA/nHA/CM scaffold (1.54 ± 0.20 MPa) was obviously higher than that of the PLGA/nHA scaffold (0.98 ± 0.12 MPa). Additionally, the compressive modulus of the PLGA/nHA/CM scaffold (29.43 ± 2.42 MPa) was significantly higher than that of the PLGA/nHA scaffold (21.45 ± 1.45 MPa). A well-designed bone-engineered scaffold has to meet two mechanical requirements to be effective. The scaffold providing a matrix for cell residence must retain structural integrity and stability when a doctor implants it into the defective site. Then, it must provide sufficient mechanical support during tissue regeneration [42]. The compression strength of the PLGA/nHA scaffold increases with the introduction of nHA [43]. Moreover, the more compressive strength of the PLGA/nHA/CMs scaffold proves that the addition of CMs can improve the mechanical properties of the scaffold without affecting the porosity.

### 3.3. Degradation Properties *In Vitro*

**Figure 5:**
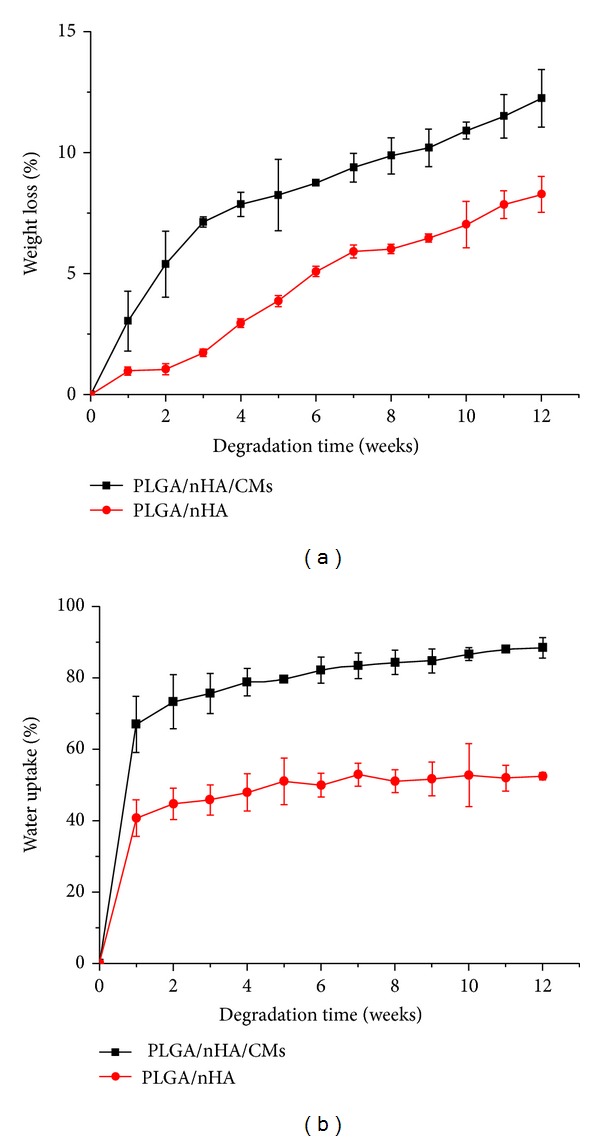
Weight loss (a) and water uptake (b) of PLGA/nHA with and without 30% CMs during degradation in PBS solution.

The degradation properties of a scaffold are of crucial importance in the long-term success of a tissue-engineered scaffold. Scaffolds for bone regeneration are designed to be gradually replaced with regenerated ECM during bone formation, accompanied with degradation. WL rate is an important parameter used to examine the degradation performance of scaffolds. The WL of the PLGA/nHA scaffolds with and without CMs is presented in Figure 5(a). The mass of the PLGA/nHA scaffolds decreased with increasing degradation time. The pure PLGA/nHA polymer showed a slower WL during the entire degradation time. The WL of the PLGA/nHA/CM scaffold was slightly faster in the first 3 weeks and then reached a linear mode. At week 12, the WL rates of the PLGA/nHA scaffolds with and without CMs were 12.23% and 8.27%, respectively. The four steps for the degradation of the PLGA scaffold are as follows: (1) swelling and hydration of the polymer, (2) breakage of the ester bonds, (3) diffusion of the soluble degradation products, and (4) disappearance of the polymer scaffold chips [44]. The faster WL detected for the PLGA/nHA/CM scaffolds can be associated to the higher capability of CMs to absorb water when soaked in PBS solution. Moreover, mass loss of the scaffolds was reported to correspond with the changes in pH [45]. The results of this study are consistent with their report. Under *in vivo* conditions, some enzymes (e.g., proteinase K and lipase PS), liposomes, germs, and phagocytes can modulate the degradation of PLGA polymers to obtain faster degradation rates. In addition, a previous study [46] reported that the faster degradation is caused by the autocatalytic effect of the acidic degradation products accumulated in the medium surrounding the implants. This effect was minimized for polymer degradation in PBS by the frequent change of the medium.

Given that medium flow is essential for nutrient and metabolic exchanges, the WA properties of a scaffold are another important feature for developing a suitable scaffold for bone regeneration [47]. The WA of PLGA/nHA scaffolds with and without CMs is presented in Figure 5(b). The WA of PLGA/nHA/CMs was 66.9% at week 1 and gradually rose stably to 82.15% at week 6, finally reaching 88.34% at week 12. The WA of the PLGA/nHA polymer was slower during the entire time and ultimately reached 52.4% at week 12. The WA result was generally consistent with the WL result. The WA of the two scaffolds rapidly increased at week 1. This increase may be attributed to the water that diffused through the porous structure. The much more rapidly increasing WA of the PLGA/nHA/CM scaffolds was possibly attributed to the excellent absorbent capacity of CMs. At the initial stage, the WA of the scaffold was critical for integrating the material-bone construct. The superior hydrophilicity of the PLGA/nHA/CM scaffolds might easily facilitate cell migration into the pores following blood immersion *in vivo*. As previously observed, the hydrophilic characteristics of the scaffolds can enhance cell adhesion, migration, and proliferation *in vivo *compared with hydrophobic scaffolds [48].

**Figure 6:**
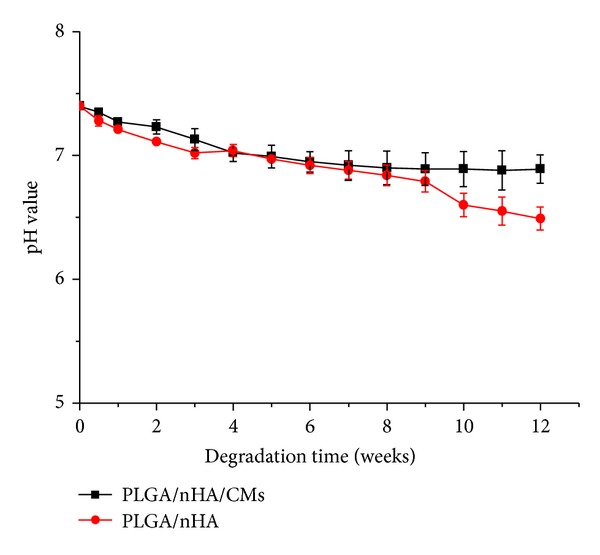
pH of incubated PBS buffer for PLGA/nHA with and without 30% CMs during *in vitro* degradation study.

The formation of the degraded acidic molecules and their release from PLGA degradation is a negative factor for bone engineering. The pH variation of PBS buffer during the degradation of the PLGA/nHA scaffolds with and without CMs was detected to verify the acid product released from the scaffold. The result is shown in Figure 6. The pH of the degradation medium generally decreased with time, maintaining nearly at approximately 7.1 until week 4 under dynamic conditions. The pH of the PBS buffer for the PLGA/nHA scaffolds with and without CMs decreased rapidly at week 4 and then slightly decreased from week 4 to week 8 of degradation. After week 8, the pH plateau was reached for the PLGA/nHA/CMs scaffold samples, whereas the pH for the PLGA/nHA scaffold samples slightly decreased. A slighter decrease in pH for the PLGA/nHA/CMs scaffolds after week 8 may be ascribed to alkaline dissolution of the CMs. Arnett [49] reported that osteoblast proliferation and collagen synthesis are unaffected by pH in the range of 7.4 to 6.9. The pH of PBS for the PLGA/nHA/CMs scaffolds was above 6.9 during the entire degradation time. This finding indicated that the composite PLGA/nHA polymer with CMs was suitable for bone engineering.

### 3.4. Gene Expression

Bone formation is an intricate and ordered cascade of synthesis of matrix proteins and calcium phosphate in a continuously renewed biological environment and regulated by a cluster of growth factors [50]. An artificial scaffold should be designed as a production of ideal structure that can mimic ECM until host cells, including both osteoblasts and vascular endothelial cells, can grow in and resynthesize a new natural matrix. The progress in replacing scaffold by natural bone is dependent on the cell adhesion, proliferation, differentiation, and vascularization of the scaffolds. Therefore, the successful formation of microvascular cells with long-term patency that are not apt to regression is very important. In bone formation, osteogenic-related genes (e.g., opn, col1, runx2, and sp7) and angiogenic-related genes (e.g., vegf and ramp2) are strictly regulated [51].

Using quantitative real-time PCR, the present study determined whether the structure of the polymer scaffold loading with chitosan-ADM microspheres and the sustained release of ADM can promote the early differentiation and activation of osteoblasts and vein endothelial cells at the mRNA level of the aforementioned genes.

**Figure 7:**
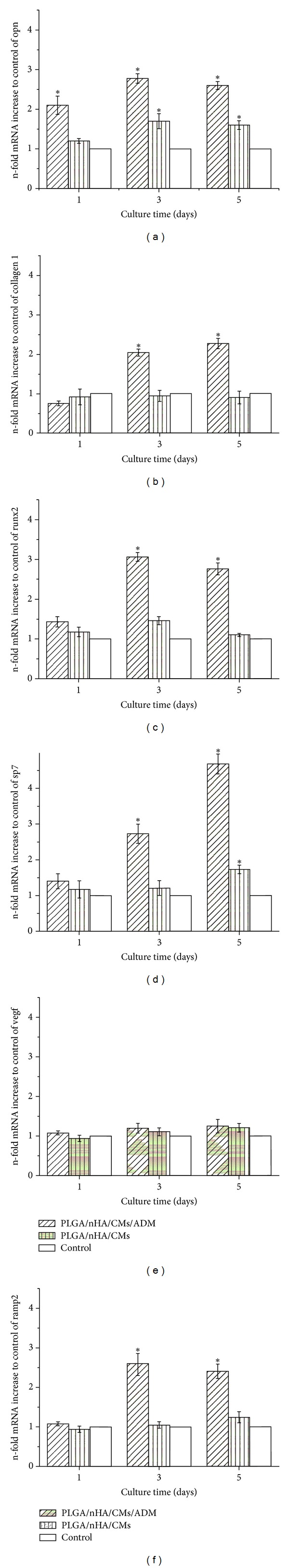
Relative mRNA expression level of MG63 cells (opn (a), collagen 1 (b), runx2 (c), and sp7 (d)) and HUVEC cells (vegf (e) and ramp2 (f)) of the PLGA/nHA/CMs/ADM, PLGA/nHA, and control groups (normal cells without treatment) at predetermined times. *P < 0.05 indicates statistically significant difference compared with the control group (*n* = 6).

On day 1, the mean expression levels of opn in the PLGA/nHA/CMs/ADM and PLGA/nHA groups were 2.1 and 1.2 times higher, respectively, than that in the control group (Figure 7(a)). On day 3, the expression levels of opn in the PLGA/nHA/CMs/ADM and PLGA/nHA groups significantly increased by 2.8 and 1.7 times higher than that in the control group. The analogous tendency appeared on day 5. Meanwhile, the opn expression of the PLGA/nHA/CMs/ADM group was higher than that of the PLGA/nHA group at each predetermined time. As shown in Figure 7(b), the col1 mRNA expression of the PLGA/nHA/CMs/ADM group was almost equal to the other two on day 1. It rapidly increased on day 3 and then maintained on day 5, which was significantly higher than that of the control group. No significant difference was observed between the PLGA/nHA group and the control group at each time point. As shown in Figure 7(c), no significant difference in runx2 expression appeared on day 1. However, on day 3, the expression of the PLGA/nHA/CMs/ADM group was 2.1 times higher than that of the control group. It then slightly decreased on day 5, which were both significantly higher than the control group. Nevertheless, as shown in Figure 7(d), the sp7 mRNA level was upregulated in the PLGA/nHA/CMs/ADM group with culture time. The values were 2.7 and 4.7 times significantly higher than those of the control group on days 3 and 5, respectively, suggesting the upregulation of osteoblastic activity. On day 5, the sp7 mRNA expression in the PLGA/nHA scaffold was 1.7 times higher than that in the control group. This result proved the bioactive potential of the interconnected microstructure of polymer scaffolds.

Cell-material interactions can be evaluated by detecting the cellular receptors responsible for adhesion and migration and the ligands they bind to, specifically bone ECM proteins [52, 53]. Our previous research revealed the proliferation and activation functions of ADM. The ADM encapsulated in the scaffold/microsphere construct can stimulate the proliferation of MG63 cells for 5 d by MTT assay and fluorescent image observation [24]. Opn, as a mineral-binding protein found in bone ECM, is implicated as an important factor in bone remodeling and crystal growth regulation. It is associated with cell adhesion, proliferation, and biomineralization of ECM into bone, and its high expression demonstrates the proliferation and activation of MG63 cells. Another ECM protein that is related to further differentiation of osteoblasts is col1, which accounts for 90% of the bone matrix proteins [54]. Frick et al. [55] reported that the mRNA expression of col1 is stimulated by alkalosis and inhibited by acidosis. The small pH variation induced by the change in calcium concentration has a significant effect on col1 expression. The increase in col1 mRNA of the PLGA/nHA/CMs/ADM group on day 3 can be due to the increase in pH induced by chitosan degradation or the effect of ADM. Transcription factors runx2 and sp7 were essential for osteoblastic differentiation and act as regulatory factors involved in osteogenic-related gene expression. Early studies reported that runx2 binds the osteocalcin promoter and is expressed in osteochondral progenitors as well as in early stages of osteoblastic differentiation [56]. In humans, runx2 haploinsufficiency results in cleidocranial dysplasia, a skeletal disorder characterized by bone and dental abnormalities [57]. Sp7-deficient mice lack bone formation with a phenotype similar to that of runx2-deficient mice. Meanwhile, sp7 is either acting downstream of runx2 or expressed later in the osteoblast differentiation pathway [58]. Based on the aforementioned result, the increase in mRNA expression at an early stage suggested that the differentiation and activation of MG63 cells on the surface of polymer scaffold were probably due to the sustained ADM release. PLGA/nHA/CMs/ADM can induce differentiation in MG63 cells at the early stage of bone formation.

The success of tissue-engineering scaffold is highly dependent on whether the materials can promote rapid and stable neovascularization (new blood vessel formation) within the scaffold, typically prior to complete material degradation [4]. Endothelial cells are currently regarded as the most interesting target for therapies aimed at enhancing or inhibiting angiogenesis [59]. VEGF is an important regulator of endothelial cell proliferation, migration, and differentiation. As one of the ADM receptors, ramp2 is essential for angiogenesis and vascular integrity. Figures 7(e) and 7(f) illustrate the vegf and ramp2 expression levels of HUVEC in three groups. The vegf expression of the PLGA/nHA/CMs/ADM group completely showed equal expression pattern to the other two groups at each predetermined time. However, on days 3 and 5, the vegf expression of the PLGA/nHA/CMs/ADM and PLGA/nHA groups was slightly higher compared with that of the control group. The ramp2 mRNA expression of the PLGA/nHA/CMs/ADM group steadily increased from day 3 and then slightly downregulated on day 5. The values of the PLGA/nHA/CMs/ADM group on days 3 and 5 were significantly higher than those of the control group. The expression level of ramp2 was rapidly upregulated from day 3 in the PLGA/nHA/CMs/ADM group, which might be induced by sustained-released ADM. However, vegf expression was not upregulated. The results of the present study are generally consistent with those of previous studies [60]. That is, the activity of ADM was triggered by the binding of ADM to its ADM receptor. In addition, the effect of ADM on HUVEC was no longer detectable after the expression of ramp2 in the cells was almost suppressed by gene silencing. The results demonstrated that the released ADM from the scaffold may be involved in vascularization from the gene level at the early stage.

### 3.5. Western Blot Analysis

**Figure 8:**
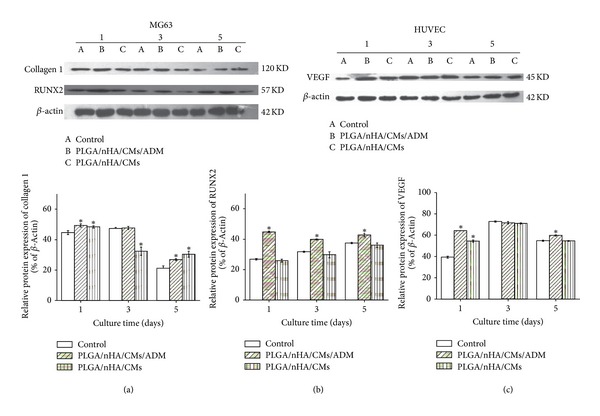
Western blot assessment of collagen 1, runx2 protein of MG63 cells, and VEGF protein of HUVEC cells in the PLGA/nHA/CMs/ADM, PLGA/nHA, and control groups (normal cells without treatment) at predetermined times. (a, b, and c) The bands were quantitated by densitometry, and data are expressed as the ratio of aim protein to *β*-actin. *P < 0.05 indicates statistically significant difference compared with the control group (*n* = 3).

To obtain further insights, COLLAGEN 1, RUNX2 protein of MG63 cells, and VEGF protein of HUVEC cells from the cell-scaffold complex were selected for detection by western blot analysis after cell culture for 1, 3, and 5 d. The results are shown in Figure 8. Significantly higher levels of COLLAGEN expression were observed in the PLGA/nHA/CMs/ADM and PLGA/nHA groups than in the control group on days 1 and 5. Moreover, significantly higher expression levels of runx2 were observed at each predetermined time on the PLGA/nHA/CMs/ADM scaffold, which was consistent with real-time PCR analysis. For VEGF, which was associated with the proliferation and differentiation of endothelial cells, the expression level of the PLGA/nHA/CMs/ADM group was significantly higher than that of the control group on days 1 and 5. Meanwhile, on day 1, the PLGA/nHA group showed significantly higher VEGF expression than the control group, suggesting that the structure and property of the PLGA/nHA scaffold were suitable for the migration and proliferation of HUVEC at the initial stage.

Western blot analysis showed higher expression of COLLAGEN 1, RUNX2, and VEGF on the PLGA/nHA/CMs/ADM scaffold during culture time. This finding suggested that composite scaffold loading with ADM promoted the differentiation and activation of MG63 cells by upregulation of the expression of specific osteogenic proteins. Higher expression levels of COLLAGEN 1 and VEGF were also observed on the PLGA/nHA scaffold. ADM has a key function during the development of the vascular system, as demonstrated by Shindo et al. [61]. Specific conditions, such as hypoxia, are reported to be associated with increased VEGF expression [62]. The ADM-induced upregulation of VEGF at the protein level agrees well with most reports [63, 64] but is not consistent with others [60, 65]. The vegf gene was not significantly upregulated. The inconsistent levels between vegf gene and protein were possibly caused by the enhancement of translational efficiency and protein constancy.

## 4. Conclusions

As a growth factor, ADM was first introduced for tissue-engineering materials. The combination of emulsion-ionic cross-linking and TIPS was proven to be suitable for scaffold/microsphere construct developing loading with ADM. The microspheres showed a rough surface morphology and were well distributed either in the presence or absence of ADM. The scaffolds showed relatively uniform pore sizes with interconnected pores. The addition of CMs into the scaffolds improved the mechanical properties of the scaffolds without remarkably changing their high porosity. Moreover, *in vitro* degradation studies revealed that CM incorporation can accelerate WL rate, increase WA, and reduce PLGA acidity in hydrolysis. The expression patterns of opn, col1, runx2, and sp7 indicated that inductive osteoblast-like cell differentiation in contact with PLGA/nHA/CMs/ADM scaffolds appeared early in bone formation. Western blot analysis demonstrated that the PLGA/nHA/CMs/ADM scaffold had high levels of COLLAGEN 1 and RUNX2 expression. In addition, RT-PCR analysis showed that the PLGA/nHA/CMs/ADM scaffold had a high level of ramp2 on days 3 and 5. The expression of vegf was unaffected regardless of ADM loading. Meanwhile, VEGF protein levels were relatively high for the PLGA/nHA/CMs/ADM scaffold at the early osteoblast stage, as determined by western blot analysis. These findings suggest that microsphere/scaffold composite was more effective in loading peptides and proteins, which can improve the osteogenic and angiogenic differentiation of osteoblasts and vascular endothelial cells on the porous scaffolds. Therefore, sustained-release ADM from microsphere-scaffold system may be a promising therapeutic agent for local application in bone tissue engineering.

## Figures and Tables

**Table 1:** Primers of genes used in quantitative real-time PCR.

Primers	Forward 5′–3′	Reverse 3′–5′
opn	GTGCCATACCAGTTAAACAG	CTTACTTGGAAGGGTCTGTG
runx2	GAGATCATCGCCGACCAC	TACCTCTCCGAGGGCTACC
Collagen 1	AGGGCCAAGACGAAGACA TC	AGATCACGTCATCGCACA ACA
sp7	CACAGCTCTTCTGACTGTCTG	CTGGTGAAATGCCTGCATGGAT
vegf	GCCTTGGTGCTCTACCTCCAC	GATTCTGCCCTCCTCCTTCTGG
ramp2	CTGCTGGGCGCTGTCCTGAA	TTCTGACCCTGGTGTGCCTGTG
gapdh	ATGGGGAAGGTGAAGGTC	TAAAAGCAGCCCTGGTGACC

**Table 2:** Density and porosity of scaffolds with and without CMs.

	Apparent density (g/mL)	Porosity (%)
PLGA/nHA	0.045 ± 0.017	90.81 ± 0.87
PLGA/nHA/CMs	0.083 ± 0.020*	88.93 ± 0.32

*P < 0.05 indicates statistically significant difference compared with the PLGA/nHA group.
